# Beyond Bench and Bedside: Disentangling the Concept of Translational Research

**DOI:** 10.1007/s10728-012-0236-x

**Published:** 2012-12-18

**Authors:** Anna Laura van der Laan, Marianne Boenink

**Affiliations:** Department of Philosophy, Faculty of Behavioural Sciences, University of Twente, P.O. Box 217, 7500 AD Enschede, The Netherlands

**Keywords:** Translational research, Translational medicine, Biomedical innovation, Conceptual analysis, Science—society relationship

## Abstract

The label ‘Translational Research’ (TR) has become ever more popular in the biomedical domain in recent years. It is usually presented as an attempt to bridge a supposed gap between knowledge produced at the lab bench and its use at the clinical bedside. This is claimed to help society harvest the benefits of its investments in scientific research. The rhetorical as well as moral force of the label TR obscure, however, that it is actually used in very different ways. In this paper, we analyse the scientific discourse on TR, with the aim to disentangle and critically evaluate the different meanings of the label. We start with a brief reconstruction of the history of the concept. Subsequently, we unravel how the label is actually used in a sample of scientific publications on TR and examine the presuppositions implied by different views of TR. We argue that it is useful to distinguish different views of TR on the basis of three dimensions, related to (1) the construction of the ‘translational gap’; (2) the model of the translational process; and (3) the cause of the perceived translational gap. We conclude that the motive to make society benefit from its investments in biomedical science may be laudable, but that it is doubtful whether the dominant views of TR will contribute to this end.

## Introduction

Translational Research—as well as Translational Medicine and Translational Science—has become a buzzword in the field of biomedical science.^1^ It shows up in the names of an increasing number of articles, reports, journals, research initiatives, research institutes and educational programmes in the domain of medical innovation. This rise went hand in hand with—and was probably partly motivated by—an increasing interest for the concept in medical research policy. Policy makers and funding institutions in several countries diagnosed a ‘gap’ that needs to be ‘bridged’ [11]. On the one side of that gap are the life sciences, producing enormous amounts of knowledge and insights about health and illness on a molecular level. On the other side there is clinical medicine, the domain that is expected to benefit from those scientific advances, but instead only sparsely progresses. ‘Translational Research’ (TR) is claimed to bridge those two sides.

A closer look at what TR in the biomedical domain is actual taken to be, however, reveals a huge diversity. Differences appear already in the short slogans that are often used to show what is TR about, such as: “Taking research from bench-to-bedside” [27]; “Bridging basic research and medical innovation” [21]; “Translating research into medical practice […]” [48]; “Translating science into better healthcare” [17]. When turning to the actual activities that are presented under the label of TR, an even more heterogeneous set passes by: the development of biomarkers, performing first clinical studies of a new technique, the marketing of a newly developed medical technology, the formulation of clinical guidelines or the integration of different types of knowledge, to name just a few.

Moreover, as several authors have noted, the label TR is not a neutral description of those diverse kinds of activities; it has a strong rhetorical as well as moral force [65, 78]. The value of TR seems to be incontestable, resulting in a “translational ethos” in biomedical science [50] or even in an obligation for biomedical researchers [19]. Performing TR seems currently the right thing to do for a biomedical scientist. Both Maienschein et al. and Denholm and Martin interpret this shift in scientific morality in terms of a change in the contract between science and society. Different from earlier generations, current citizens and governments distrust public investments in science. They want value for money, and TR is supposed to increase the chance of achieving such value. Since the plea for TR carries such a strong normative force, it is worthwhile to reflect on what ‘Translational Research’ is taken to mean in the biomedical domain, and how it is supposed to contribute to public benefit. In this paper we want to provide such reflection.

Up to now, the phenomenon of TR has received remarkably limited attention from the social sciences and humanities. There is some literature on ethics and TR, mainly focusing on research ethical issues [35, 39, 73]. In addition, there are a few sociological studies of TR, often analysing interactions between different actor groups involved in TR [13, 43, 49, 60, 78]. In both cases, usually a specific concept of TR is either implicitly or explicitly taken as a starting point. However, as indicated above, the meaning of TR should not be taken for granted. As some authors suggest, an in depth qualitative study of the various manifestations of TR is needed to better understand the ethos of TR [66].

In this article we want to take a broader as well as a more analytical view. We start with a brief reconstruction of the history of the concept ‘translational research’ in the biomedical domain. Subsequently, we unravel how the label is actually used in biomedical scientific discourse. We argue that it is useful to disentangle different views of TR by distinguishing three dimensions, related to (1) the construction of the ‘translational gap’; (2) the model of the translational process; and (3) the cause of the perceived translational gap. We will critically discuss the presuppositions of the positions found for each dimension. Finally, we will ask how plausible it is that the conceptions of TR dominant in the scientific literature will indeed help society to harvest benefits from biomedical science.

### Translational Research in the Biomedical Domain: Its History in a Nutshell

**Fig. 1:**
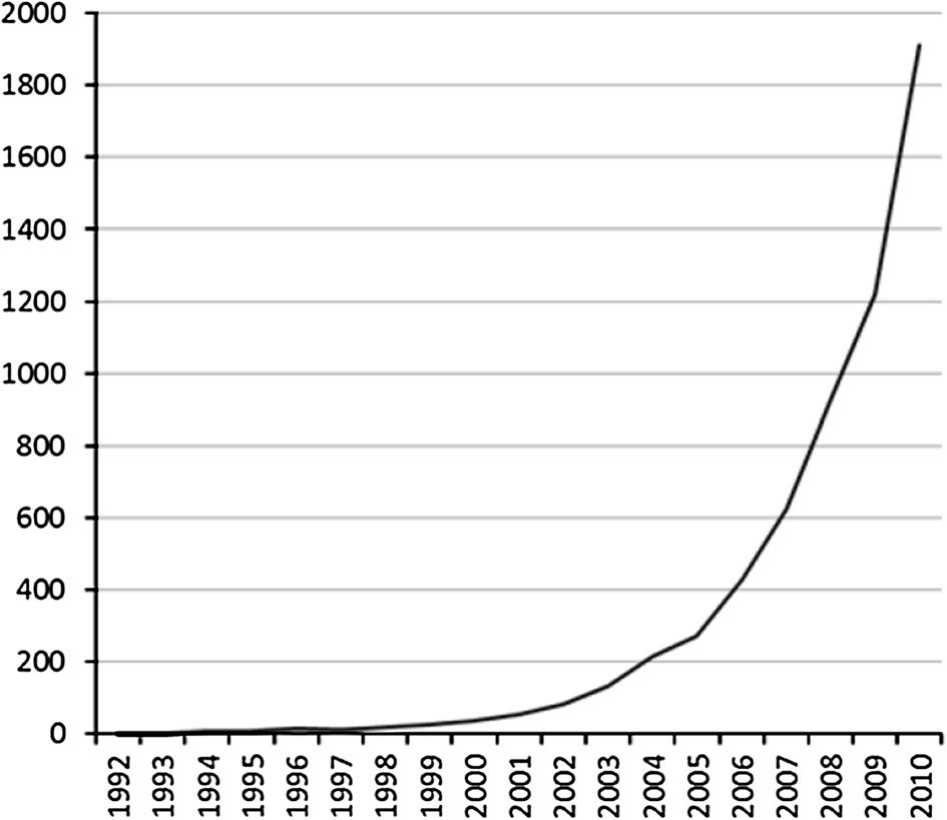
Number of published articles with the terms “Translational Research”, “Translational Science” or “Translational Medicine” each year, as appeared in a Pubmed search (carried out September 9, 2011)

As a simple search for the term ‘translational research’ in Pubmed illustrates,^2^ the label appeared for the first time in biomedical journals around 1993. Only from about the year 2000 onwards the number of medical-scientific publications on TR starts to grow exponentially (see Fig. 1).

All early publications using the term deal with cancer research (see for example: [10, 54, 56, 57, 62, 83]. This can be attributed to the Specialized Programs of Research Excellence (SPOREs), established by the U.S. National Cancer Institute (NCI) in 1992. In response to growing concerns that scientific knowledge was not always directly and effectively applied to reduce the burden of cancer, these programs encouraged TR for different kinds of cancer [54, 58]. The SPOREs were supposed to:

promote interdisciplinary research and to speed the bi-directional exchange between basic and clinical science to move basic research findings from the laboratory to applied settings involving patients and populations [58].

The NCI also was the first to link the term TR to the phrase ‘from bench to bedside’, as it can be seen in the following definition of TR by NCI director Broder and his colleague Cushing:

Translational research moves knowledge about cancer in either direction, between findings at the laboratory bench and clinical observations at the bedside. Both preclinical and clinical research are translational if the specific goal is to move the fruits of basic knowledge closer to clinical application [10].

After 1993, then, the term started to pop up in biomedical articles on cancer research. The label at this time was used, for example, for research on the discovery and validation of biomarkers as promising surrogate endpoints for cancer prevention research [57]. Using biomarkers was argued to have great potential for accelerating and reducing the costs of developing effective cancer prevention strategies. Around 1997, the label TR for the first time appeared in the name of cancer research centres in Canada and North America. Such centres sought to facilitate TR by starting tissue banks and by creating alliances between researchers working on different stages of cancer research, as well as between scientists and private partners. In subsequent years, the term was taken up outside the USA and Canada, and also in other disciplines, such as AIDS research [32], cardiology [9] and gastroenterology [69]. TR slowly lost its unique association with cancer research and was increasingly put forward in all areas of biomedical research.

A crucial turning point in the history of TR is the year 2003, when the American National Institutes of Health presented the Roadmap for Medical Research. This Roadmap claimed to address the “critical scientific gaps” blocking the transformation of discoveries in the life sciences into improvements in health [84]. In subsequent years, other countries took similar initiatives. For example, the British Medical Research Council (MRC) started to show interest in TR, understood as research *“*accelerating the translation of fundamental research into benefits for the person, in health and disease*”* [53]. In the Netherlands, the Advisory Council on Health Research (RGO—Dutch acronym) wrote a report in which the strengths and weaknesses of the Dutch research system with regard to TR are mapped. The RGO describes TR as: *“*a phase in the knowledge chain. It comprises all steps from the identification of possible leads (in patients or patient material) for diagnostics, prevention or treatment, up and including early application in clinical practice. Research questions may originate from clinical practice as well as from the laboratory*”* [1]. And in 2009 the EU established the European Advanced Translational Research Infrastructure in Medicine (EATRIS), in which ten European countries participate [4, 21]. EATRIS aims to *“*provide infrastructure to allow a faster and more efficient transfer of research discoveries into new products to prevent, diagnose or treat diseases*”* [21].

From 2003 on, then, TR has appeared high on the policy agenda for biomedical research in a growing number of countries. However, by then the motivation to stimulate TR seems to have slightly shifted. The first TR-initiatives were driven by the desire to finally see effective treatment for an awful disease. The more recent initiatives start from the observation that health improvements have not kept up with the increased speed of discovery in the life sciences, such as genomics and molecular biology. The completion of the Human Genome Project (still quite recent then) definitely played a role here. After all, this lavishly funded ‘big science’-project did not bring about the promised revolutionary changes in health care or the expected health gains. Recent pleas for TR are thus partly rooted in disappointment about the societal profit from basic research.

Whatever its motivation, the popularity of TR in research policy circles clearly affected the biomedical research domain. Research centres were set up, journals were launched, research infrastructures were built and educational programmes were developed, all devoted to TR. It is no surprise, then, that the number of scientific articles on TR grew exponentially (see Fig. 1). However, the quantitative picture is misleading. Looking beyond the label itself, it quickly becomes visible that TR is interpreted in very different ways. To evaluate what the rise of the label TR actually means, we should first disentangle its different meanings.

## Methods

Since the literature on TR is booming, we analysed a sample of documents. We focus on the biomedical scientific literature, and not on the science policy documents, because the first shows how the label is used by those most directly involved in doing TR. Science policy documents, in contrast, reveal more about ideals than about actual activities. We started with the set of literature identified by the PubMed search described above. We limited the results first by selecting those publications published in ‘core clinical journals’. These journals have been indexed by the U.S. National Library of Medicine as high quality clinical English language journals. From this set, we further selected those publications in which the term TR (or Translational Science/Translational Medicine) is used in the *title* of the publication.^3^ This resulted in a set of 107 articles published between 1993 and 2011. The collection comprises a mixture of editorials and commentaries in which the concept of TR is more or less explicitly reflected upon, and research articles in which results and findings of so-called ‘TR’ are reported.

We analysed this sample asking two questions. First, since translation implies that one thing is translated into something else, what are the domains supposed to be in need of communication? And secondly, which activities are thought to achieve translation? We found that a huge variety of activities, by very different actors in different practices, is labelled ‘TR’. Subsequently, we identified three dimensions that are helpful to distinguish different views of TR. First, it is useful to ask how the perceived ‘translational gap’ is actually constructed. Secondly, the translational process is modelled in very different ways. Finally, the perceived cause(s) of the translational gap deserve attention. We will discuss each dimension and the different positions found on that dimension in the subsequent sections.

### Which Translational Gap?

The metaphor of translation suggests that TR deals with at least two domains: A is translated into B. But *what* is in need of translation? And *into what* should it be translated? Or, to phrase it differently, what are the two sides supposed to constitute the gap in need of a bridge?

One side of the gap (let’s call it side 1) is identical in most publications on TR. It is referred to with terms like ‘new knowledge’ (also: ‘research findings/discoveries/ideas/insights/information’) gained from ‘basic (biomedical) research’ (also: ‘animal studies/in vitro studies/pre-clinical studies’). Although the range of scientific disciplines and research methods supposed to yield such knowledge slightly varies, the general idea remains the same: the knowledge to be translated comes from pre-clinical studies, in which the human body was not yet involved.

The variety of interpretations of TR appears only when the other side (side 2) of the translational gap is pointed out. We identified *six* variants for side 2 in the documents we consulted. The different constructions of the gap are described in Table 1, illustrated by a characteristic quote.

**Table 1:** The construction of translational gaps

Side 1	Side 2	Example
Results from new knowledge gained from basic scientific research	A) New approaches or methods	“[…] translating understanding into entirely new approaches to therapy” [6]
	B) Knowledge of the human body	“[…] investigations in humans which define the biology of disease” [55]
	C) Medical applications	“[…] to translate more quickly the myriad discoveries in biomedical research into more effective applications relevant to human health and disease” [19]
	D) Improvement of clinical practice	“[…] to improve the application of scientific discoveries from “the bench” to actual patient care at “the bedside” […]” [22]
	E) Benefit for the individual patient	“[…] translation of […] scientific knowledge into patient benefit […]” [25]
	F) Improvement of public health	“From evidence based medicine to sustainable solutions for public health problems” [47]

This overview shows that the gap TR is supposed to bridge can be constructed in rather narrow as well as quite broad ways. Many documents stick to a narrow conception, in which TR should bridge a gap between basic science on the one hand, and new approaches for pre- clinical work, knowledge of the human body or medical applications on the other. The gap in these cases is situated *within R&D practice*, or rather between many different R&D practices. The conceived result of TR is more (different) knowledge, or a new technology. A smaller, but not negligible number of publications wants TR to address a much wider gap, between basic science on the one hand and clinical practice or the actual health condition of individuals and populations on the other. In these cases, the gap spans beyond R&D practices, pointing out that innovative knowledge and products are not automatically used once available. This difference between narrow and broad conceptions of the translational gap has been noticed before, and is sometimes indicated by distinguishing T1 (narrow) and T2 [82] or even T1 (narrow) and T2, T3 and T4 (different varieties of broad TR) [38].

When discussing TR, then, one should be aware that there is not just one translational gap; there are many gaps. Clearly, what TR is supposed to do and aim for depends on the way this gap is constructed. For any publication on TR it makes sense to question which gap TR is actually supposed to address. The broader the gap, the more complex and multifarious TR becomes. In the broader views TR is not just about translating one type of knowledge into another kind of knowledge, as is more or less the case when side 2 is identified with A or B. It can also be about translating knowledge into (better) technologies, practices, bodies (or persons), and social statistics. With each subsequent broadening of the gap, the potential obstacles to translation increase in kind and number, and achieving translation will therefore take more and different types of efforts. Bridging the broader gaps requires that practices and habits have to be reshaped and that roles and responsibilities of various actors have to be changed. Accordingly, a broad construction of the translational gap tends to interpret TR in an extremely comprehensive as well as ambitious way.

Defining the gap in a narrow way, as most of the documents in our sample do, has definite advantages. It implies a relatively unambiguous and maybe more feasible conceptualization of TR. However, the broader constructions of the translational gap rightly remind us that the value of new biomedical knowledge or of a new biomedical technology lies in its effects on our health. The additional activities included in the broader conceptions of TR therefore do not replace, but are added to the activities of the narrower views: they complete the picture of what is necessary to harvest the promised values (usually health and quality of life, but there may be others as well). When TR is conceptualized in a narrow way, it risks to loose sight of what is necessary to make knowledge or applications valuable for individual users and society. This risk is indeed visible in the documents we studied. Repeatedly, the objectives of a publication are framed in a broad way, whereas the endpoints chosen in the described research address narrow gaps only. Stüve et al. [75] for example, review the advances in ‘translational research’ in the area of multiple sclerosis. A narrow view on TR becomes visible: the review centralizes the need for good animal models to evaluate newly developed therapies. Still, it is argued that such models “will ultimately benefit patients” (p. 1312)—reflecting broader aims of TR. The same can be seen in Lainka et al. [42], in which the term TR refers to efforts of setting up integrative databases and international research networks in order to get a better understanding of auto-inflammatory diseases. Despite this narrow and rather specific use of the term of TR, the authors argue that their work will allow “translation of newly discovered pathogenic mechanisms […] into improvements in patient care […]” (p. 241). These examples implicate a lack of attention for further work needed to harvest the potential value of their research. In that sense, references to the term TR are in danger of becoming a facile self-justification. Similar mismatches are even more problematic in research policy, when huge budgets are spent on narrow translational research, whereas activities addressing the broader variants of the translational gap are neglected.

### Models of the Translational Process

**Fig. 2:**
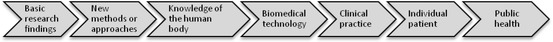
Translational research as a linear process of different succeeding steps

The second dimension useful to distinguish different interpretations of TR is the underlying model of the translational process. Here three positions can be recognized: a linear view, a bi-directional, and a complex view. A majority of the literature on TR seems inspired by a linear model of innovation. TR is then seen as a sequence of steps, in which realization of the last aim (individual or public health) is dependent on realizing the preceding ones. Many authors distinguish different steps or phases in TR [28, 31] or speak of a “TR continuum” [7, 20]. The different steps could be represented as follows (Fig. 2).

The underlying idea is that innovation starts with basic science. The knowledge that results from basic science is then supposed to be translated in ideas and knowledge about real (diseased) bodies and in medical technologies, that have to be implemented in clinical practice. This will then result in healthier individuals and improved public health.

Such a linear model of innovation is of course well known and ubiquitous in discourse about innovation, both in R&D, science policy making and economics [26]. It has definite advantages, because it orders the different stages of innovation on a timescale and suggests a clear division of labour, thus indicating a seemingly logical way to organize work on future innovation. However, it has also been disqualified as empirically inadequate. Stokes [74] for example, discusses the inadequateness of the commonly held distinction between basic and applied science and their supposedly temporal, linear relationship. He points out that something like “use-inspired basic research” had a significant role in the history of biomedical innovation. This type of scientific research is driven by anticipated use right from the start. He mentions the work of Louis Pasteur, which substantially contributed to both fundamental understandings in microbiology and advances in the treatment of bacterial diseases, as an example of such use-inspired research. In addition, Bijker [8] shows how social groups ‘pull and push’ [34] the process of technology development in various directions, which is difficult—if not impossible—to predict. In retrospect, then, innovation may appear to be a linear process, but such a reconstruction disregards that “the “successful” stages in the development are not the only possible ones” [64].

The linear model is not only empirically inadequate to describe innovation processes, however. Several studies show that the division of labour and the timing suggested by the linear model, when used prospectively, may actually *block* successful innovation. A lack of attention for implementation, regulatory or ethical issues during early phases of innovation may cause failure in implementation or lack of acceptance [24, 44]. Views of TR that conceptualise translation as a linear, unidirectional process thus risk to be unfruitful or even counterproductive.

Although a linear view of the translational process is dominant, a substantial subset of the documents we analyzed stresses that translation should go in two directions. In fact, the conception of TR as a two-way road was already brought forward in the NCI definition cited above. Many authors argue that a unidirectional view on translation neglects that basic science and technology development can learn from and integrate findings from experiments, clinical studies, and even from knowledge from clinical practice and from population studies. They claim that ‘backwards translation’ is necessary.

This ‘backwards translation’ can be conceptualized in a narrow or a broad(er) way. Narrowly defined it means that results from knowledge produced in a specific stage of research (like clinical trials) has to be ‘fed back’ to earlier phases of the research process (like lab research) [14, 37]. The type of information translated backwards is limited to scientific knowledge and translation takes place within the R&D domain. This narrow conception of backwards translation is reminiscent of the ‘empirical cycle’ of research, in which scientific results inspire new hypotheses for subsequent projects.

Documents advocating broader approaches of backwards translation argue that early phases of innovation should also account for knowledge related to clinical practice, individual and/or population behaviour. The range of proposals with regard to whose and which knowledge to communicate includes: issues ‘relevant to routine care delivery’, provided by primary care clinicians [22]; knowledge derived from clinical practitioners and community settings [28]; results from observational studies, epidemiology studies, social and behavioral sciences, and cost-effectiveness studies [45, 52] and patient preferences [31]. There are also pleas to engage end-users [29, 33, 67] and communities [68, 86] in establishing research agendas and priorities, in study design and in the whole innovation trajectory. Some authors even go beyond the image of two way traffic and characterize the translational process as ‘multidirectional’, ‘iterative’ and ‘dynamic’, thus stressing its complexity [19, 29, 52]. The translational process is then regarded as a continuous data exchange within and between various research and non-research practices.

The idea behind proposals for backwards translation in a broad sense, as well as the multidirectional and dynamic views of the innovation process, is that ‘translation’ will be more successful when researchers account for information from practices, actors and communities that may be affected by the products of R&D. On the one hand, backwards translation may improve the future translatability of new knowledge and innovations by anticipating relevant features of its context of use (whether the human body, clinical practice or patients’ lives). This will make innovations more *useable* for the projected users. On the other hand, backwards translation may help to figure out early how innovations will best fit the needs and values of those they affect. This makes innovations more *relevant* to the projected users.

Backwards translation, in particular the broad version, can thus be a valuable extension of the linear model still guiding a lot of thinking on TR. It may correct the misguiding division of labour and timing suggested by this model, which can lead to ineffective innovation. That being said, in the literature we analysed it often is not very clear what type of knowledge is actually to be translated backwards and how an effective translation could be achieved. Patients, for example, are often considered to have ‘experiential knowledge’ of their disease, their body and how to live with the disease. It is not obvious, however, how such knowledge can be taken up in, say, molecular research. At this point, more work is definitely needed.

### The Cause(s) of the Translational Gap

Whatever the domains in need of translation, and whatever the direction(s) translation is supposed to take, something has to be *done* to achieve translation. In the set of documents we analysed two sorts of activities can be distinguished. First of all there are activities like funding, creating new venues for publication, or changing regulation. These activities are assumed to realize the conditions necessary for translation—they *facilitate* TR. Second, TR is also identified with activities directly related to doing biomedical research; doing research in the strict sense of designing and performing experiments to test formulated hypotheses. Accordingly, two groups of perceived *causes* of the translational gap (whatever its construction) are identifiable: ‘external’, non-scientific causes and ‘internal’ causes found in biomedical scientific practice itself. Of course, explanations for the translational gap need not be mono-causal; many documents put forward a number of factors contributing to the translational gap. For the sake of clarity, however, we will discuss these two groups separately.

Some argue that TR is a new label referring to ‘ancient practices’ [72]. And indeed, in our set of documents the label TR is frequently used to refer to standard types of biomedical research like clinical trials, interventional studies or observational studies [5, 36, 41]. In cases like these, the research necessary for translation is nothing new—it is research ‘as usual’. The dominant view in the documents we analysed is that TR does not denote new research approaches, but existing types of research in need of some sort of external support. The shared diagnosis in these cases is that the translational process is obstructed by many *non*-scientific hurdles. These may concern all kinds of practical, social, economic or ethical obstacles, like: lack of funds for expensive clinical trials, lack of communication between lab researchers and clinicians, or strict regulation for research with human subjects, making researchers shy away from the administrative work involved. An array of measures is proposed to address these ‘external’ blocks and thus to stimulate TR. Which measures are singled out depends on the characterization of the translational gap and the translational process. Most frequently, the translational gap is localized within the R&D-domain, within a uni-linear process of innovation. The proposed measures then are, among others: removing (ethical) regulatory obstacles [59], changing recruitment of research subjects [81], new ways of recruiting and training researchers [70], stimulating collaboration between academy and industry [16], stimulating communication between researchers and clinicians [71] and publishing preliminary research data in journals [46].

In contrast, when the translational gap is localized between research and clinical practice (D), other causes and activities are stressed. For example, lack of professional awareness of the state of the art of biomedical sciences is argued to hinder translation [23], and developing clinical guidelines is proposed as an essential translational endeavour [18]. When the gap is located between implementation and improved health (E and F), it is for example mentioned that expenses related to the use of a new drug impede translation, which urges insurance companies to change their reimbursement policies [76]. Lack of *backwards* translation is also frequently linked to external causes, for example because of restricted access to results of scientific research [3]. The same goes for the general lack of interest of medical journals for negative results [77]. For this reason, most new journals in translational research have an open access policy and claim to publish both positive and negative results. It is also frequently argued that research to produce clinically relevant knowledge, such as clinical trials and observational research, receive insufficient attention and funding [82]. From that perspective, there is hardly anything to translate backwards in the first place.

All views identifying only external causes for the translational gap imply that translational research in itself is not particularly difficult. It is not performed as frequently as it should be, because this part of scientific work is expensive, time or energy consuming. As a result, few people are willing to do the job. The point of TR according to these views is to make translational work easier and more attractive by removing unnecessary obstacles.

In addition to the very prevalent ‘externalist’ view, some documents identify science itself as the main cause of the translational gap. In these cases, translation is supposed to be difficult or impossible because scientific knowledge is not easy to translate, and this in turn is thought to be due to the methods used in science. For example, several authors argue that in vitro and animal models produce a type of knowledge that does not adequately represent the complex mechanisms of the human body [15, 51]. They argue that more complex animal models or models of human clinical tissue are needed [55, 61], because these produce better—more ‘translatable’—knowledge.

Other authors find fault with randomized clinical trials [18, 47, 80], because these often produce knowledge that is valid for a very specific group of patients only. From this perspective, the translational gap is at least partly created by the artificiality of experimental set ups. This artificiality sits badly with the complexity of real word settings and is expressed in the possible tension between internal and external validity [2, 28, 80]. To overcome this cause of the translational gap, then, we need biomedical research methods that use ‘real world’ settings and outcome measures [80]. The use of biomarkers as surrogate endpoints is also frequently mentioned as part of TR [30, 40, 63]. They can function as a ‘translator’ between bodily processes and the symptoms of patients, and thus help to save time and costs of clinical trials with clinical endpoints.

In documents identifying ‘internal’, scientific causes for the lack of translation, TR is anything but ‘science as usual’. As NIH director Zerhouni argues, we need “truly novel approaches to and methodologies for conducting research” [86]. In addition to producing more realistic research models and using real world data, a recurrent theme is the need for convergence or (re-)integration: of different life sciences [3], of different experimental approaches [6], of life sciences and clinical sciences [79] and even of life sciences and all kinds of population studies [16]. Such integration requires computational research with large databases of molecular, clinical and epidemiological information. Better information technology systems are therefore assumed to be a critical condition for TR—indicating that these novel research methods, like the old ones, should be facilitated [12, 16, 85].

The views of TR starting from an ‘internalist’ explanation of the translational gap, then, are geared towards the production of better knowledge by doing *innovative science*. The resulting knowledge is supposed to be more ‘translatable’, that is, easier to take up in other domains, both within science (for example when using better animal models) or in clinical practice and beyond (for example by using patients’ expertise to develop a new technology). Backwards translation can be an important driver to develop innovative research methods, in particular when including non-scientific expertise in discussions on the research set up.

To sum up this section: the documents we analysed identify factors both internal and external to science as causes for the lack of translation. Of course, the boundary between science and its context is highly porous as well as contested, so any demarcation of ‘internal’ and ‘external’ is liable to criticism. We use the terminology here simply to distinguish causes related to scientific methods and the knowledge they produce from causes that do not put into question the methods and products of current scientific work. This distinction is relevant because it attributes roles and responsibilities to specific actors. Whereas the external views tend to select policy makers, funders, teachers and trainers as actors who can further the translational process, the internal views mainly look at scientific researchers and technology developers. To be sure, many authors mention more than one cause, and propose a mix of activities by a broad array of actors to achieve translation.

It seems indeed plausible that the lack of translations (in plural) has multifactorial origins. If this is true, the ‘externalist’ views predominating our set of documents have important deficiencies. These views tend to take the translatability (including the relevance and usefulness) of the knowledge produced by science too much for granted. The ‘internalist’ views rightly point out that the translational gap is at least partly created by science itself. It makes sense, then, to stimulate innovative ways of science, but also to create the financial, organizational and regulatory conditions for doing so.

## Conclusion: A New Contract Between Science and Society?

We started our analysis by observing that ‘translational research’ quickly has become an imperative in biomedical science. The plea for TR seems driven by a changed relationship between science and society. ‘Translation’ has become important because society is thought to deserve a tangible return (in terms of health and quality of life) on its investment in (often basic) biomedical science. We also pointed out, however, that the meaning of TR should not be taken for granted. Even though the normative justification remains similar, different views of TR circulate.

Our analysis of the scientific discourse on TR shows that it is indeed useful to disentangle different views of TR. For each document on TR, it is useful to ask what its position is on three dimensions. The dimensions and the possible positions are summarized in Table 2.

**Table 2:** The dimensions of translational research

Dimension	Positions
Scope and location of the translational gap	Narrow
	Broad
Model of translational process	Linear
	Bi-directional
	Complex
Cause of the translational gap	External to science
	Internal to science

The first dimension concerns the scope and the precise location(s) of the translational gap. Narrow views of TR focus on a perceived gap in science or in medical practice; broader views aim to address several of the narrow gaps in one (albeit complex) move. The narrow view is dominant. Although the rather ambitious broader view has definite weaknesses, it does clearly point out that the dominant narrow view of TR takes too much for granted. New medical knowledge or new medical technologies in themselves are usually not enough to realize the values of health and quality of life.

The second dimension regards the supposed translational process. Most frequently, the translational process is supposed to be uni-linear, proceeding from lab research to clinical trial to practices of use. However, quite a few authors contest the implied assumption that knowledge or technologies once produced will automatically be taken up by the target group. They argue that some form of *backwards translation* should be stimulated. We also identified more *complex models* picturing TR as an iterative, multidirectional and dynamic process. Both in the bi-directional and the complex views the usability and relevance of medical knowledge and technologies is thought to be crucial for an effective translation process.

The third dimension deals with the perceived cause of the translational gap. The dominant ‘externalist’ views focus on removal of obstacles for doing science. They propose measures to facilitate scientific research that would help lab knowledge move forward, but that scientists are reluctant to take on. The ‘internalist’ views argue that science itself is part of the problem, and suggest that innovative ways of producing knowledge and technology are necessary. These views rightly point out that the translatability of scientific results may be assumed too easily, and that scientific methods may widen or even create a gap with clinical practice and the world outside the lab.

The positions on the three dimensions are often interrelated. Although all combinations might be possible in principle, in practice some combinations occur more often than others. It is important, moreover, to note that the different views do not necessarily exclude each other: more than one translational gap may exist, and the cause for each gap may be multifactorial. This means that opting for a specific view of TR is more an issue of priority setting in a specific context than of judging truth. It is quite clear, however, that some positions are dominant. The view of TR we encountered most frequently in the documents we studied

1. identifies a narrow gap within R&D practice,
2. assumes a linear model of innovation, and
3. locates the causes of the gap outside science.

As a result, TR is equated with phase I or II clinical trials, and the activities proposed to stimulate TR aim to facilitate the step from animal studies to such trials.

How does this dominant view relate to the normative ideal driving TR? Is this indeed a good way to ensure that society will benefit from its investments in biomedical science? On the basis of our analysis, we sincerely doubt this. For a start, by focusing on the gap between lab/animal research and clinical trials this view takes for granted that knowledge from clinical trials will ultimately lead to benefit for individuals and/or the public at large. Secondly, by working with a linear model of the translation process it misses the opportunity to find out what (groups in) society consider as benefits, inquiring which knowledge or technology is thought to be relevant and usable. It also does not take advantage of expertise of potential users or stakeholders. And finally, it does not look critically at the ways in which science itself may impede translation. As a result, even if this view of TR would succeed in stimulating more clinical trials, the knowledge produced may not lead to tangible benefits because it does not fit with the intended users’ characteristics, practices and, most importantly, values.

The more marginal views of TR rightly stress the importance of

1. explicit attention for the implementation of innovations in practice,
2. backwards translation and iterative innovation processes to ensure that the resulting knowledge or technology is relevant and useable to intended users, and of
3. a careful crafting of scientific methods to connect lab and ‘real life’ situations as tightly as possible.

Actually, these aspects all indicate how important it is to anticipate ‘what comes next’: if biomedical R&D should contribute to benefit for individuals and the public, it had better continuously ask how decision making during current stages of innovation can best reflect the characteristics and values of future users and stakeholders. This will enhance the ‘translatability’ of the knowledge produced, as well as the motivation of subsequent actors to take up this knowledge in their own work.

More generally, our analysis serves as a reminder that the attempt to make society benefit from its investment in biomedical science is laudable, but also quite ambitious. To realize it, many different activities, by different actors, in different settings need to be performed, to cross many different gaps. It is quite misleading/confusing to label all these activities and contributions with one label, TR, because it suggests that it would suffice to perform and stimulate one type of research. It may be more helpful to think of such beneficial innovation processes in terms of a nexus (or web) of many *translational moments*: moments at which the design of present work needs to anticipate, and to be coordinated with, the requirements and characteristics of its future contexts of use. A guarantee that public funding for biomedical science will lead to benefits for society is impossible to give, but continuously and consciously connecting decision making during R&D to the future use of its results may be our best shot for harvesting such benefits.

## Footnotes

1 As far as we can judge on the basis of the literature studied, these labels are used interchangeably in the biomedical domain. Although our study includes documents using all three terms, for purposes of brevity we will use the term ‘Translational Research’ only. To be clear, whereas this term might be used in other domains as well, our analysis is limited to the way these terms are used in the biomedical domain.

2 The PubMed search was carried out with the following terms: "translational research"[All Fields] OR "translational medicine"[All Fields] OR "translational science"[All Fields]. We filtered our search by data, which up to “1992/12/31” yielded no hits. From 1993 onwards we noted the hits per year by adding “AND "YEAR/01/01"[PDAT] to "YEAR/12/31"[PDAT]”. Carried out September 9, 2011.

3 Search details: "Translational Research"[Title] OR "Translational Medicine"[Title] OR "Translational Science"[Title] AND jsubsetaim[text]. Carried out December 19, 2011.
